# Does Nanosilver Exposure Modulate Steroid Metabolism in the Testes?—A Possible Role of Redox Balance Disruption

**DOI:** 10.3390/biomedicines12010073

**Published:** 2023-12-28

**Authors:** Michał Oczkowski, Katarzyna Dziendzikowska, Joanna Gromadzka-Ostrowska, Michał Rakowski, Marcin Kruszewski

**Affiliations:** 1 Department of Dietetics, Institute of Human Nutrition Sciences, Warsaw University of Life Sciences (WULS-SGGW), Nowoursynowska 159C, 02-776 Warsaw, Poland; katarzyna_dziendzikowska@sggw.edu.pl (K.D.); joanna_gromadzka-ostrowska@sggw.edu.pl (J.G.-O.); 2 Cytometry Laboratory, Department of Oncobiology and Epigenetics, Institute of Biophysics, Faculty of Biology and Environmental Protection, University of Lodz, Pomorska 141/143, 90-236 Lodz, Poland; michal.rakowski@edu.uni.lodz.pl; 3 Centre for Radiobiology and Biological Dosimetry, Institute of Nuclear Chemistry and Technology, Dorodna 16, 03-195 Warsaw, Poland; m.kruszewski@ichtj.waw.pl; 4 Department of Molecular Biology and Translational Research, Institute of Rural Health, Jaczewskiego 2, 20-090 Lublin, Poland

**Keywords:** oxidative stress, rats, redox balance, silver nanoparticles, steroid receptors, testes

## Abstract

Silver nanoparticles (AgNPs) are a popular engineered nanomaterial widely used in industry. Despite the benefits they bring to society, AgNPs are not neutral to human health. The aim of this study was to evaluate the effects of a single intravenous dose (5 mg/kg body weight) of 20 nm AgNPs on steroid metabolism and redox balance in the testes of adult rats. The effects were evaluated 1 day or 28 days after intervention and compared with saline-treated animals. Decreased aromatase and estrogen receptor α levels (by 21% and 27%, respectively) were observed 1 day after AgNPs administration, while increased testosterone, increased dihydrotestosterone levels, higher androgen receptors and higher aromatase expression in Leydig cells (by 43%, 50%, 20% and 32%, respectively) as well as lower (by 35%) androgen receptor protein levels were observed 28 days after exposure to AgNPs compared to control groups. The AgNPs treatment resulted in decreased superoxide dismutase activity, decreased GSH/GSSG ratio, and increased glutathione reductase activity (by 23%, 63% and 28%, respectively) compared to control animals, irrespective of the time of measurement. Increased (by 28%) intratesticular lipid hydroperoxides level was observed 1 day after AgNPs exposure, while decreased (by 70%) GSH and increased (by 43%) 7-ketocholesterol levels were observed 28 days after treatment compared to control animals. Conclusions: AgNPs exposure caused redox imbalance in the gonads shortly after AgNPs administration, while a longer perspective AgNPs exposure was associated with impaired androgen metabolism, probably due to increased oxidative stress.

## 1. Introduction

Silver nanoparticles (AgNPs) are one of the most popular engineered nanomaterials widely used in industry, from the manufacture of electronics, textiles and consumer goods to applications in healthcare, agriculture and the food production sector [1]. Over the past two decades, the use of AgNPs has grown tremendously due to their antibacterial, antifungal and antiviral properties. While the growth of industrial and biomedical applications of AgNPs brings undeniable benefits, in vitro and animal model studies have shown potentially harmful effects of AgNPs on human health. Nanosilver can cross biological barriers, accumulate in tissues and exert a wide range of adverse effects on the liver, brain, testes and other organs [2,3].

The intravenous route of nanoparticles administration allows them to bypass the gastrointestinal or respiratory tract and enter directly into the bloodstream, from which they can be distributed to different organs [4]. Therefore, the systemic delivery of nanoparticles improves the identification of toxic mechanisms aimed at target cells and tissues [5]. Additionally, intravenous administration is a well-known model for studying the effects of nanoparticles used for diagnostic and therapeutic purposes when they should have access to all organs and tissues in the body [6,7]. The results reported by Dziendzikowska et al. [8] showed that the same treatment regimen used in this work resulted in accumulation of AgNPs in different tissues in a time-dependent manner. The biological activity of AgNPs is determined by their properties, such as size, shape, surface potential or functionalization [9]. However, the harmful effects of AgNPs in cells result from their chemical activity. After uptake, AgNPs are usually targeted to lysosomes, where the acidic environment and aerobic conditions promote the release of silver ions (Ag^+^) and the formation of Ag-O- moieties. Both forms of silver have the ability to decrease mitochondrial membrane potential and stimulate the formation of reactive oxygen species (ROS) [10]. Results from in vivo experiments investigating the distribution and toxicity of nanoparticles have shown that intravenous injection of AgNPs induces an oxidative stress response in primary organs (liver and spleen), but the pro-oxidative effects of NPs exposure can also be observed in other organs [11,12].

Although the gonads are not a primary target organ for nanoparticles accumulation, gonadotoxic effects may be similar to those observed in the liver, kidney or spleen, leading to dysfunction and misregulation of the reproductive system. Negative effects of exposure of rats to AgNPs have already been reported [13]. In a continuation of previous work, the present study focused on the effects of intravenous administration of AgNPs on the steroid-hormone signaling pathway and redox balance in rat testes. The effects of a single dose (5 mg/kg of body weight) of 20 nm AgNPs were evaluated 1 day or 28 days after administration.

## 2. Materials and Methods

### 2.1. Silver Nanoparticles Preparation and Characterization

In the in vivo experiment, animals were exposed to spherical AgNPs of 20 ± 5 nm in diameter coated with bovine serum albumin (BSA) purchased from PlasmaChem (Berlin, Germany). The preparation of AgNPs was detailed in previous works [14,15]. To summarize, 2 mg of AgNPs were dispersed in 800 μL of purified distilled water, forming the nanoparticle stock solution. The stock solution was sonicated for 10 min on ice using a probe sonicator (Branson, Danbury, CT, USA) with a total ultrasound energy of 420 J/m. After sonication, 100 μL of 15% BSA and 100 μL of 10× phosphate-buffered saline (PBS) were directly added to the solution. Additionally, the aggregation state of AgNPs was examined alongside the assessment of its zeta potential and hydrodynamic size. The characterization was conducted using scanning electron microscopy (DSM 942, Carl Zeiss, Göttingen, Germany) and transmission electron microscopy (JOEL 1200 EX II, JOEL, Tokyo, Japan). The outcomes of these analyses have previously been published [14]. The details pertaining to the AgNPs used in the in vivo experiment can be found in Table 1.

### 2.2. Animals and Treatment

The animal experiment was performed on fourteen-week-old male Fischer 344 rats (Fischer 344/DuCrl) with an initial body weight of 244.2 ± 10.7 g, purchased from Charles River, Sulzfeld, Germany. During the experiment, the animals were individually housed in polyurethane cages under stable environmental conditions (temperature 23 °C, 60% humidity, air change rate 15 times/h and 12:12 h artificial light–dark photoperiod). The animals had free access to water and feed (Sniff^®^ Spezialitäten GmbH R/M-H, Soest, Germany). The experimental procedures were approved by the 3rd Local Ethical Commission in Warsaw (Resolution No. 35/2014). They were performed in accordance with the UE Directive (2010/63/UE), the Polish legal regulation, and with respect to the 3R rules.

**Figure 1:**
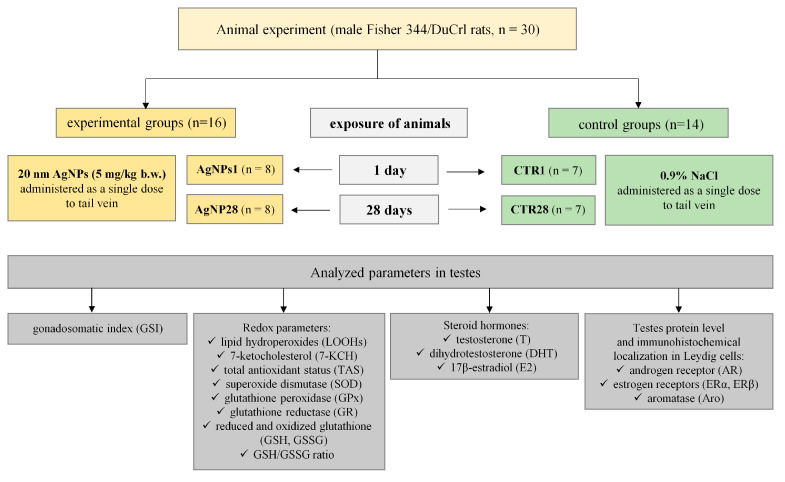
The experimental design of the in vivo experiment.

After 10 days of acclimatization, the rats were divided into two experimental (AgNPs1 and AgNPs28, *n* = 8 each) and two control (CTR1 and CTR28, *n* = 7 each) groups. The animals from experimental groups were administered 20 nm AgNPs (5 mg/kg b.w.) as a single dose intravenously into the tail vein and euthanized after 1 day (AgNPs1) or after 28 days (AgNPs28). Control groups (CTR1 and CTR28) were treated with 0.9% NaCl solution using the same volume and route of exposure. The animals were euthanized under deep Isoflurane (Baxter Healthcare, Warsaw, Poland) anesthesia and sacrificed via heart bleeding, after which both testes were dissected, weighed and rinsed with ice-cold PBS. The left testis was immediately frozen in liquid nitrogen and stored at −80 °C until further analysis, while the right testis was fixed in formalin and used for histological and immunohistochemical analyses. The scheme of the in vivo experiment is presented in Figure 1.

### 2.3. Determination of Intratesticular Redox Parameters

Left testis tissue samples were homogenized on ice in 50 mM PBS (pH = 7.0) with 1 mM EDTA (1:10 *m*/*v*) using a Bio-Gen PRO 200 homogenizer. The homogenates were centrifuged (10,000× *g*, 15 min, 4 °C) and supernatants were aliquoted for subsequent analysis. The lipid hydroperoxides (LOOHs) and 7-ketocholesterol (7-KCH) levels were analyzed as oxidative stress parameters in testes. To evaluate the level of LOOHs, 100 μL of testis homogenate supernatant was measured colorimetrically in duplicate using the method described by Yagi [16]. The absorbance of the sample was read at 665 nm after incubation at 30 °C for 10 min. A standard curve was generated using 50 nmol/mL cumene hydroperoxide. The 7-KCH level in the testes was analyzed in duplicate using high-performance liquid chromatography with UV detection (HPLC-UV, Beckman Counter Inc., Brea, CA, USA) according to the method described in detail in our previous paper [17].

The activities of superoxide dismutase (SOD), glutathione peroxidase (GPx) and glutathione reductase (GR), as well as total antioxidant status (TAS) in the testes, were measured in order to assess the testes antioxidant potential. TAS concentration and activity of SOD, GPx and GR were determined using Randox (Randox Laboratories Ltd., Crumlin, Co., Antrim, UK) commercial kits (catalog numbers: NX2332, SD125, RS504, GR2368, respectively) according to the protocols provided by the manufacturer. The concentration of reduced (GSH) and oxidized (GSSG) forms of glutathione in the testicular supernatants were determined via HPLC, as described in [18], using a 4-channel electrochemical array for simultaneous detection. All samples were analyzed in duplicate and the GSH/GSSG ratio was calculated.

### 2.4. Determination of Concentration of Testicular Steroid Hormones

The steroid hormones (androgens: testosterone [T] and dihydrotestosterone [DHT]) and 17β-estradiol [E2]) were extracted from the supernatants of the testes using an equal volume of ethyl acetate saturated with water. The mixture was vortexed twice; the organic phase was collected and dried under nitrogen at 37 °C. The residue was dissolved in PBS buffer containing 0.01% bovine serum albumin (BSA) [2,19]. Hormones concentrations were determined via the enzyme-linked immunosorbent (ELISA) method, using commercial kits from Demeditec Diagnostics GmbH (Kiel, Germany; catalog numbers: DE1559, DE2330 and DE4399 for T, DHT and E2, respectively) according to the manufacturer’s instructions. All samples were analyzed in duplicate.

### 2.5. Determination of Estrogen Receptors (ERα ERβ), Androgen Receptor (AR) and Aromatase (Aro) Protein Concentrations

The testicular tissue samples were homogenized in cold PBS with protease inhibitor cocktail. After two cycles of freezing and thawing (at 4 °C), the samples were centrifuged at 5000× *g* (4 °C), and the supernatants were used for further analysis. The concentrations of steroid receptors (ERα, ERβ, AR) and aromatase (Aro) were evaluated using rat-specific ELISA kits (catalog numbers: 1050r, 2300r, 1252r and 2098r, respectively) purchased from EIAab^®^ (Wuhan, China). The tissue homogenates were prepared according to the ELISA kits manufacturer’s instructions. All samples were analyzed in duplicate.

### 2.6. Immunohistological Evaluation of Estrogen Receptors (ERα, ERβ), Androgen Receptor (AR) and Aromatase (Aro) in Leydig Cells

Immunolocalization of steroid receptors and aromatase in Leydig cells (LCs) was performed on 6 μm thick sections. A detailed description of the method has been published previously by our team [20]. Briefly, sections were washed in PBS (pH = 7.4) and incubated overnight at 4 °C with polyclonal anti-rabbit primary antibodies (catalog numbers: sc-816 from Santa Cruz Biotechnology, Paso Robles, CA, USA, for AR; NCL-L_ER-6F11 from Novocastra, Newcastle, UK, for ERα; 07-359 from Millipore, Darmstadt, Germany, for ERβ and MCA2077S from AbD Serotec, Oxford, UK, for Aro). After extensive washing in PBS, the samples were incubated with biotinylated goat anti-rabbit IgG secondary antibody (Cat. No. 830 from Immunotech, Marseille, France). The samples were incubated with 3% H_2_O_2_ to inactivate endogenous peroxidase activity and then with peroxidase-conjugated streptavidin (catalog number: 309 from Immunotech, Marseille, France). The 3′,3′-diaminobenzidine (DAB; Sigma-Aldrich; St. Louis, MI, USA) was used as a detection system. The results of staining were examined with a standard Olympus BX41 light microscope and expressed quantitatively as a weighted average (Id score), which was calculated as the sum of the intensity of the staining multiplied by the percentage of stained LCs [21], according to the formula Id score = (N1 × 0 + N1 × 1 + N2 × 2 + N3 × 3)/100. The Id score ranged from 0 (no staining in all cells) to 3 (cells with strong staining intensity).

### 2.7. Statistical Analysis

**Figure 2:**
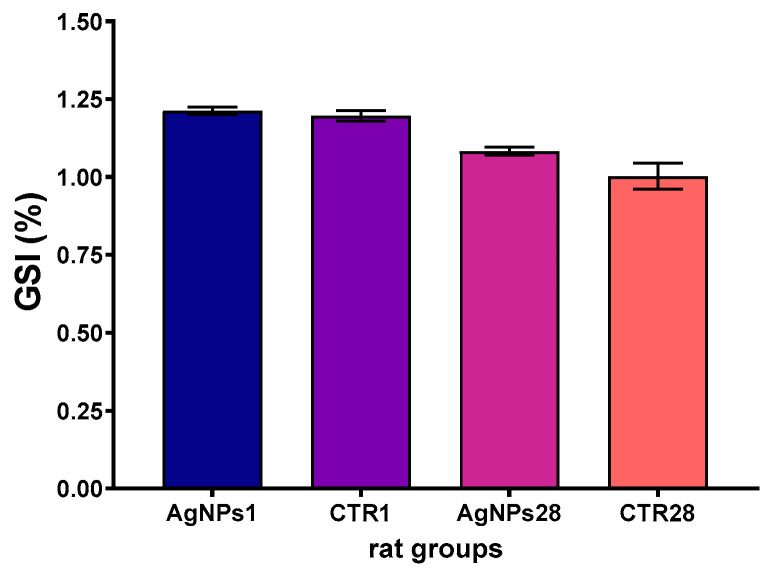
Gonadosomatic index (GSI) in the testes of rats following 1 day (AgNPs1 group) or 28 days (AgNPs28 group) after intravenous administration of AgNPs. CTR1 and CTR28—corresponding control groups. Data are expressed as mean ± SEM. Differences between treated and corresponding control group are not statistically significant.

**Figure 3:**
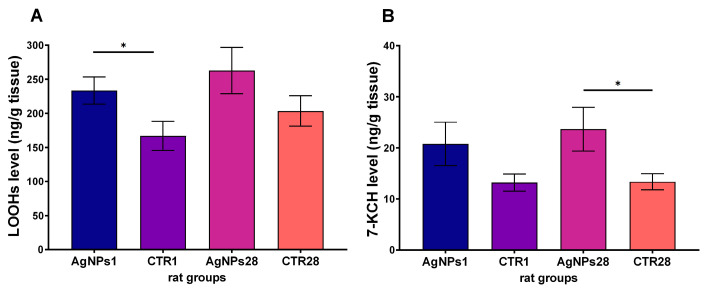
Lipid hydroperoxides (**A**) and 7-ketocholesterol (**B**) levels in testes of rats following 1 day (AgNPs1 group) or 28 days (AgNPs28 group) after intravenous administration of AgNPs. CTR1 and CTR28—corresponding control groups. Data are presented as mean ± SEM. One-way ANOVA with Fisher’s post hoc test. *—*p* < 0.05.

**Figure 4:**
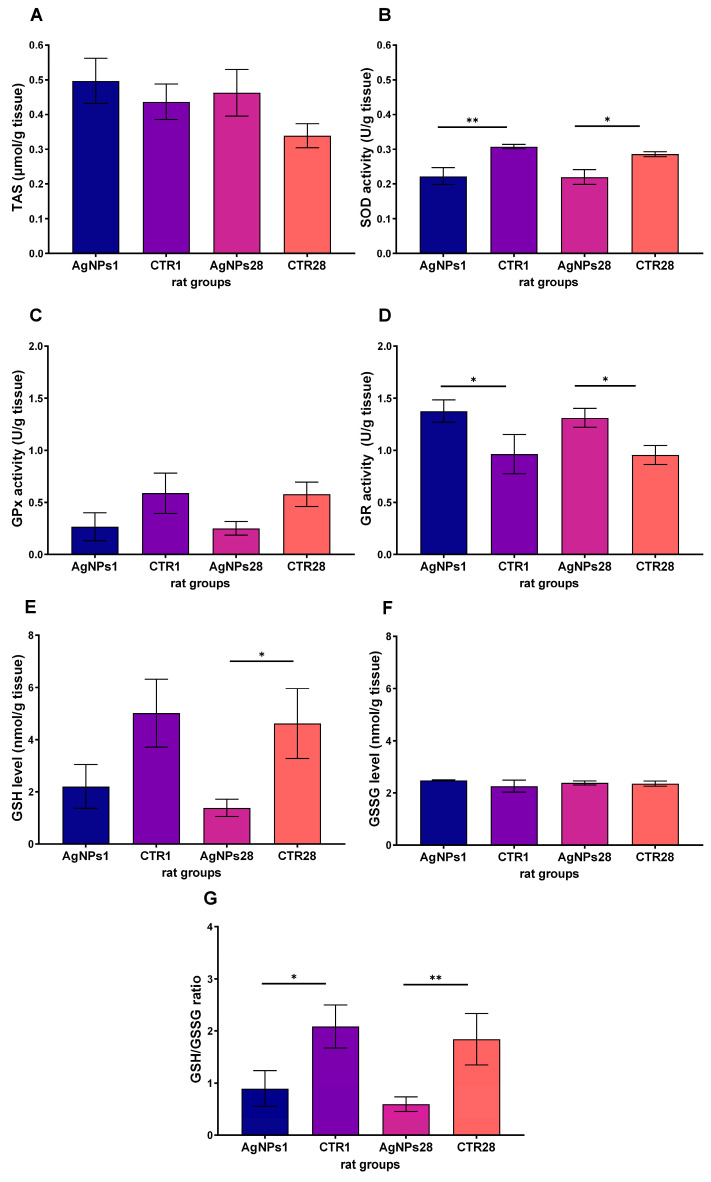
The level of total antioxidant status (TAS) (**A**), the activities of superoxide dismutase (SOD) (**B**), glutathione peroxidase (GPx) (**C**), glutathione reductase (GR) (**D**) activities, the level of reduced (GSH) (**E**) and oxidized (GSSG) (**F**) form of glutathione and GSH/GSSG ratio (**G**) measured in rat testes 1 day (AgNPs1) or 28 days (AgNPs28) after intravenous administration of AgNPs. CTR1 and CTR28—corresponding control groups. Data are presented as mean ± SEM. One-way ANOVA with Fisher’s post-hoc test. *—*p* < 0.05; **—*p* < 0.01, one-way ANOVA with Fisher’s post-hoc test.

**Figure 5:**
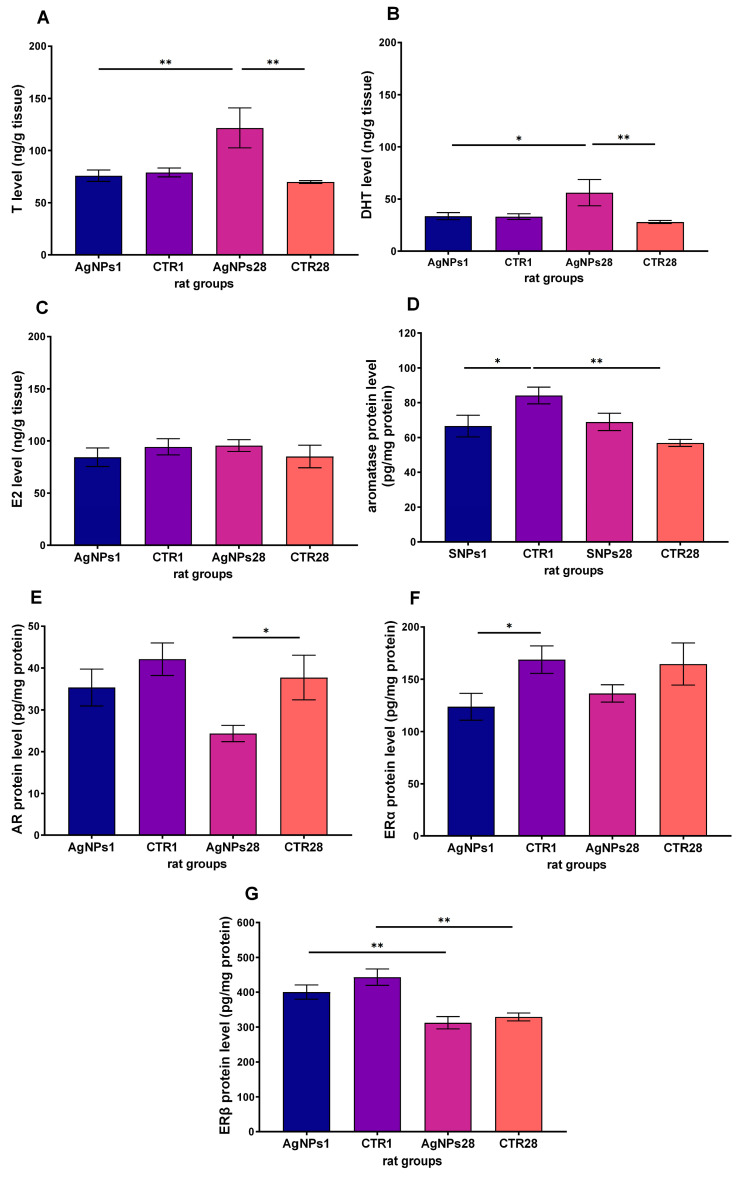
The level of steroid hormones (testosterone (**A**), dihydrotestosterone (**B**), 17β-estradiol (**C**)), as well as aromatase (**D**) and steroid receptors (androgen receptors (**E**), estrogen receptor α (**F**) and estrogen receptor β (**G**)) measured in rat testes 1 day (AgNPs1) or 28 days (agNPs28) after intravenous administration of AgNPs. CTR1 and CTR28—corresponding control groups. Data are presented as mean ± SEM. One-way ANOVA with Fisher’s post-hoc test. *—*p* < 0.05; **—*p* < 0.01, one-way ANOVA with Fisher’s post-hoc test.

**Figure 6:**
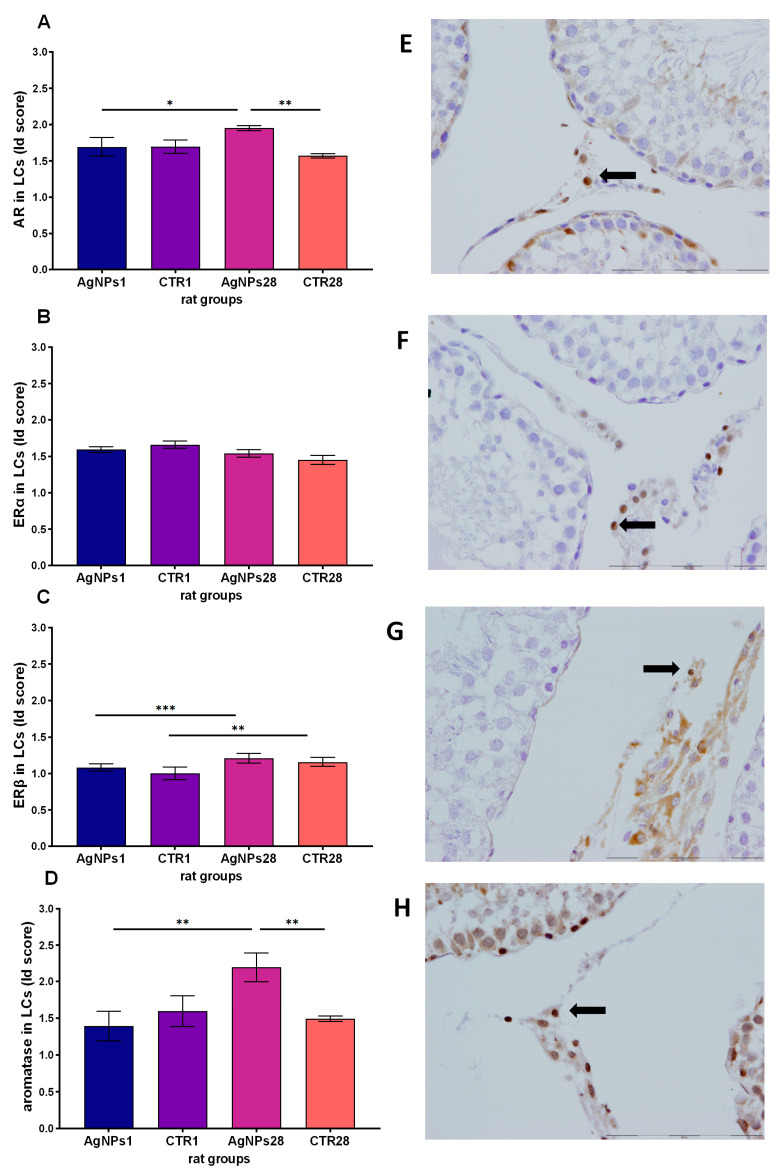
Androgen receptor (**A**), estrogen receptors α (**B**) and β (**C**) and P450 aromatase. (**D**) Id score in Leydig cells (LCs) measured in rat testes 1 day (AgNPs1 group) or 28 days (AgNPs28 group) after intravenous administration of AgNPs. CTR1 and CTR28—corresponding control groups. Data are presented as mean ± SEM; * *p* < 0.05, ** *p* < 0.01, *** *p* < 0.001, one-way ANOVA with Fisher’s post-hoc test. Immunostaining of androgen receptors (**E**), estrogen receptors α (**F**) and β (**G**) and aromatase (**H**) in rat LCs (400×). The black arrows indicate a positive staining in LCs.

**Figure 7:**
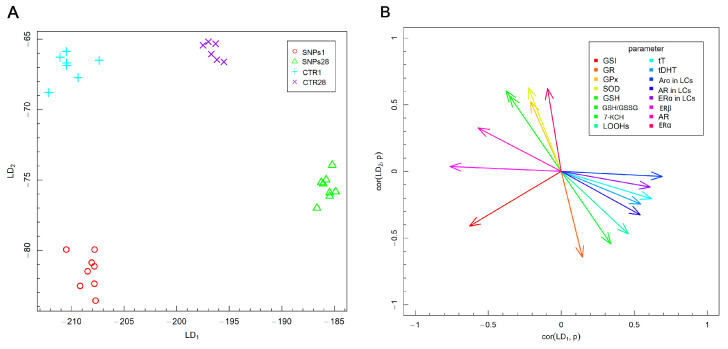
Fisher’s linear discrimination analysis: (**A**) experimental data on the plane spanned by the two the most data separating LDs; (**B**)—the parameters contributing the most to LDs; t for testicular; LCs for Leydig cells.

Statistical analysis was performed using Statistica software ver. 13.3. All data were analyzed via one-way analysis of variance (ANOVA) with the post hoc Fisher test. Statistical significance was set at *p* < 0.05. All results were expressed as mean ± SEM (Standard Error of Mean). Figure 2, Figure 3, Figure 4, Figure 5 and Figure 6 were drawn using GraphPad Prism ver. 9.2.0 ans Figure 7 was drawn using R statistical software v. 4.2.3. Fisher’s linear discriminant analysis (F-LDA) was performed using R statistical software v. 4.2.3. (https://cloud.r-project.org/ (accessed on 21 February 2023)) (R: The R Project for Statistical Computing) using the MASS package [22]. The interaction between investigated parameters was also analyzed at the two time points.

## 3. Results

### 3.1. Overall Animals’ Condition, Body Weight Gain and Gonadosomatic Index

All rats ate steadily and showed no clinical signs of adverse health conditions. The final body weight (278.4 ± 4.7 g in the AgNPs28 group and 286.8 ± 4.7 g in the CTR28 group) and total body weight gain (47.2 ± 1.4 g and 51.0 ± 3.1 g, respectively) were not different among all groups.

The gonadosomatic index (GSI) value did not differ in the AgNPs1 and AgNPs28 groups, as compared to the corresponding control groups. The observed differences in GSI were due to changes in body weight of animals after 4 weeks of the experiment (Figure 2).

### 3.2. Male Gonads Redox Parameters

One day after receiving a single injection of AgNPs, an increased level of LOOHs in the testes was observed in comparison to the control rats (*p* < 0.05), as depicted in Figure 3A. This trend was also observed 28 days after administration of AgNPs, but the difference was not statistically significant due to the higher level of LOOHs in the control group. Additionally, the level of 7-KCH was found to be elevated after the administration of AgNPs, but the difference was only statistically significant 28 days after injection (*p* < 0.05), as shown in Figure 3B.

Further analysis indicated no significant alterations in TAS level in the rats’ testes (Figure 4A) or GSSG concentration (Figure 4F). Although GPx activity was lower in the treated groups, the differences were not statistically significant (Figure 4C). On the other hand, the activity of SOD exhibited a decrease in the testes of rats exposed to AgNPs both 1 and 28 days after AgNPs administration (Figure 4B), whereas the activity of GR was higher in both groups (Figure 4D). The concentration of GSH decreased in the exposed groups (Figure 4E), while GSSG concentration did not change (Figure 4F); thus, GSH/GSSG ratio also decreased (Figure 4G) in both exposed groups, as compared to appropriate controls.

### 3.3. Steroid Hormones Concentration in Testes Homogenate Supernatant

The concentration of both androgens in the testes, T and DHT (as shown in Figure 5A and 5B, respectively), significantly increased 28 days after administering AgNPs in comparison to the values obtained 1 day after nanosilver exposure and in the corresponding control group. However, there was no significant difference in testes level of E2 between the groups (as shown in Figure 5C). The level of Aro protein (Figure 5D) was significantly lower just 1 day after AgNPs injection (*p* < 0.05), likely due to the significant reduction in its activity in the 28-day control group, as there was no significant difference between the AgNPs1 and AgNPs28 groups. The level of androgen receptor protein (depicted in Figure 5E) significantly decreased 28 days after AgNPs treatment. Meanwhile, the level of the ERα protein (Figure 5F) was significantly lower 1 day after AgNPs injection. The concentration of ERβ protein (Figure 5G) was higher 1 day after injection of AgNPs than 28 days after, possibly resulting from the significant decrease in the ERβ protein level in the control group (CTR28).

### 3.4. Steroid Hormones Receptors and Aromatase Protein in Leydig Cells

Statistically significant changes were observed in the presence of four proteins analyzed in Leydig cells (LCs) of the studied groups. The AR Id score value (Figure 6A) was observed to be higher in testes secretory cells sampled 28 days after AgNPs injection than in the control group (*p* < 0.01) and in the group sampled 1 day after injection (*p* < 0.05). No difference in Id score between groups was found with regards to expression of ERα receptor. Similarly, ERβ Id score in LCs (Figure 6C) did not significantly differ between AgNPs-treated groups and appropriate control animals. The highest Id score value in LCs was observed in the case of aromatase (Figure 6D). As with androgen receptors, Id score 28 days after AgNPs treatment was significantly different from Id score for the group assessed 1 day after NPs injection and for the 28-days control group (*p* < 0.01 in both cases).

Fisher’s linear discriminant analysis (F-LDA), presented in Figure 7, was performed to examine the differences in the analyzed parameters related to AgNPs systemic exposure among animal experimental groups. The F-LDA was used to optimize the linear combination of oxidative stress and antioxidative defense parameters, as well as sex steroid metabolism parameters, to obtain the best separation of the experimental groups. The data depicted in Figure 7A demonstrate the linear combinations of parameters (linear discriminants, LDs), marked as LD1 and LD2, which consecutively separate the best experimental groups. Additionally, Figure 7B demonstrates the correlation between the two LDs and the analyzed parameters.

The AgNPs-treated animals were separated from the control animals, irrespective of the time points following AgNPs exposure (Figure 7A), based on (1) the level of testicular T and DHT; (2) the activity of GR, GPx, SOD and GSH levels, as well as GSH/GSSG ratio; (3) the level of 7-KCH and LOOHs and (4) estrogen and androgen receptors, as well as Aro concentration. The control groups of rats were characterized by higher SOD activity and GSH levels in the testis (Figure 4B and 4E, respectively), which mainly allowed them to be separated from the experimental groups. The AgNPs-treated groups were differentiated on the basis of significance of the LD1 coefficient, which was correlated with the GSI and levels of steroid hormones receptors—AR and ERβ, as well as the level of Aro protein (positively correlated with LDA1) (Figure 7). The animals from the group exposed to AgNPs and sampled 1 day after injection in F-LDA analysis were also separated from those sampled after 28 days. The position of those groups on the graph was determined mainly by the levels of steroid hormones and their receptors, including both estrogen receptors and AR protein levels and proteins involved in their metabolism (Figure 5E–G). Animals from the group exposed to AgNPs and sampled 1 day after injection presented a higher level of AR, ERβ and Aro protein. In contrast, rats sampled 28 days after AgNPs injection were characterized by increased DHT level, which primarily caused a split of AgNPs-treated groups in F-LD analysis. Summarizing, the F-LDA results indicate that separation of the control groups and treated ones was based mainly on the parameters of oxidative stress and antioxidant defense. Meanwhile, separation of treated groups was possible via parameters reflecting the metabolism of steroid hormones in gonads.

The results of the experiment indicate that intravenous administration of a single dose of AgNPs caused changes in rats’ testes, primarily related to a disturbed redox balance resulting from increased oxidative stress and impaired antioxidant defense. On the other hand, the concentration and localization of steroid hormones and their receptor proteins underwent changes over time following exposure to AgNPs, with greater impact observed 28 days following injection. These findings are summarized in Table 2.

## 4. Discussion

The concern regarding the potential health effects of AgNPs is supported by the results of toxicological studies. Our study aimed to evaluate the impact of a single intravenous nanosilver injection on steroid metabolism in the testes of adult male rats, with a focus on the role of oxidative stress as a possible mechanism responsible for AgNPs toxicity. Our results show that exposure of rats to nanosilver caused various changes in the male gonads, including alterations in redox balance and steroid receptors levels, which were observed 1 or 28 days after a single intravenous exposure.

Intravenous application of AgNPs, as used in this study, allows direct contact of the tested agent (AgNPs) with peripheral circulation and mimics potential AgNPs exposure for biomedical purposes. The nanomaterial is rapidly transported to various organs and passes through blood–tissue barriers, such as blood–testis barrier (BTB).

Our results show that even a single intravenous administration of AgNPs caused time-dependent changes in steroid metabolism in the testes. The differences in observed endpoints at 24 h and 28 days after administration might result from differences in AgNPs deposition and redistribution. Dziendzikowska et al. [8] reported that, 24 h after a single intravenous dose of AgNPs to male Wistar rats, nanosilver was predominantly deposited in the liver. However, 7 days after administration, the highest level of silver was observed in the lungs, while 28 days after AgNPs administration, the highest silver deposits were observed in the kidneys and brain. Although the silver concentration in testes was not assessed, it can be postulated that silver concentration in the testes might increase over time, similarly to the brain, due to the barrier separating both organs from the rest of the body. While blood–brain and blood–testis/epididymis barriers differ histologically, their functions are similar. This is due to the specificity of the gene phenotypic features of endothelial cells and differences in tight and adherens junction protein expressions that restrict the passage of molecules between the organ’s lumen and outer space [23,24,25].

Animal health monitoring during the experiment indicated no adverse effects of AgNPs injection on daily food intake and total body weight gain. These findings align with those reported by Xue et al. [26], who investigated the AgNPs toxicity and bio-kinetics of AgNPs (7.5 to 120 mg/kg bw/day for 8 weeks) in mice. Our study mainly indicated time-dependent changes in GSI and showed lower relative testes weight 28 days after AgNPs administration. These findings are in line with our previous results from an in vivo study and suggest that even a single administration of AgNPs may have a long-term effect on the testes [13].

The severity of the effects increased with time elapsed since AgNPs injection, as shown by the results regarding steroid metabolism. Changes in the testicular steroid metabolism were more significant 28 days following AgNPs administration compared to 1 day after, with an increase in concentration of androgens (T and DHT), a decrease in expression of the androgen receptor and an increased immunohistochemical Id score value of androgen receptors and aromatase in LCs. Fisher’s linear discriminant (F-LDA) analysis confirmed an increased level of DHT in the testes 28 days after administration of AgNPs. Similar effects on steroid secretion were reported in mice exposed to 1 mg/kg b.w. of 10 nm citrate-coated AgNPs [27]. The mechanism responsible for a higher level of testicular androgens may be linked to hypertrophy of Leydig cells and the concomitant up-regulation of genes involved in steroidogenesis in testes, such as Cyp11a1 (cholesterol side-chain cleavage enzyme, P450scc) and Hsd3b1 (3-β-hydroxysteroid dehydrogenase). The effectiveness of androgen biosynthesis in LCs depends on cholesterol transport to the mitochondria. Furthermore, P450scc protein synthesis plays a crucial role in the synthesis of both pregnenolone and testosterone. Several authors reported that the Cyp11a1 gene may increase extra-gonadal tissues following oxidative stress induced by NPs [28]. In contrast to these findings, Dziendzikowska et al. [3] demonstrated that a single intravenous administration of 20 nm AgNPs resulted in a significant decrease in Cyp11a1 and Hsd3b1 gene expressions and lowered testosterone and DHT levels in testes, compared to control animals 28 days after a single exposure. As previously discussed, AgNPs have adverse effects on gonadal steroidogenesis, resulting in decreased testosterone synthesis. These effects were likely due to increased apoptosis of LCs [29] or impaired transport of cholesterol into the LCs’ mitochondria [30].

In the current study, decrease in intratesticular Aro protein level was observed shortly after exposure to AgNPs (1 day). The findings are in line with those obtained by Han et al. [31], who showed a decrease in Cyp19a1 gene expression in mice exposed to 20 nm AgNPs (1 mg/kg b.w.). In contrast, Dziendzikowska et al. [3] reported no change in Aro protein level in male gonads 1 day following AgNPs exposure and a reduced enzyme level 28 days after 20 nm AgNPs (5 mg/kg b.w.) exposure. The differences in Aro activity observed in this study, compared to those reported by Dziendzikowska et al. [3], may be partly attributed to a specific stage of the seminiferous epithelium (SE) cycle, during which male gametes mature. Spermatogenesis is a complex process, divided into 14 stages in rats, and the time required for male gamete maturation is 52 days [32]. Bois et al. [33] demonstrated a wide range of variability in Aro gene expression level depending on the stage of SE cycle.

It is evidenced that estrogens play a crucial role in regulation of male reproductive function. Specifically, at the testicular level, these hormones are predominantly produced in LCs (in adult subjects) and control spermatogenesis. Additionally, they also inhibit the effects of luteinizing hormone, thereby reducing testosterone synthesis [34]. The impact of estrogen in target cells is mediated via nuclear estrogen receptors (ERα and β) signaling pathways. Referring to the results of this study, there were no observed changes in the expression of both estrogen receptors in the testes LCs either 1 or 28 days after AgNPs administration, with the exception of a decrease in ERα protein level in the testes 1 day after administration. This suggests that a single treatment of AgNPs only has a temporary impact on ERα signaling in the testes. However, the decreased Aro protein level in the testes, as observed in our study, alongside the absence of changes in the testicular E2 level shortly after AgNPs injection, hints towards the possibility of peripheral E2 aiding in the prevention of decreased E2 level in the gonads.

Our study’s findings suggest that steroid metabolism changes in the testes were associated with redox balance both 1 and 28 days after administration of AgNPs. A surge of oxidized lipids and increased GR activity were observed shortly (1 day) and longer-term (28 days) after single AgNPs exposure, along with a reduction in SOD activity, GSH level and the ratio of both glutathione forms. In addition, no changes in GSSG levels or GPx activity were observed after AgNPs administration. Silver nanoparticles present in both blood and cells have the potential to release Ag^+^ and bind to GSH thiol groups, forming Ag–GSH complexes [35]. This in turn can lead to reduced levels of GSH. Following the intravenous administration of a single dose of AgNPs, the nanoparticles accumulate primarily in the liver and subsequently release Ag^+^ into the bloodstream for up to one month, eventually reaching target organs such as the testes. The prolonged persistence of Ag^+^ may also lead to a decrease in GSH levels. Furthermore, the lack of changes in GPx activity observed in our study suggests that AgNPs may not be involved in the generation of H_2_O_2_. Abu-Taweel et al. [36] also reported decreased SOD activity in mice that received repeated intraperitoneal administration of 20 nm AgNPs (40 mg/kg once a week for 5 weeks). The accumulation of AgNPs in the testes typically leads to reduced GSH concentration and decreased activity of enzymes involved in antioxidant defense, such as SOD and catalase, resulting in higher ROS production in germ and somatic cells in the gonads [37].

Increased production of ROS and impaired redox parameters down-regulate estrogen signaling, leading to impaired spermatogenesis [38]. It is important to emphasize that ROS are by-products of normal testicular cells metabolism, and oxidative stress at a physiological level is necessary to regulate many functions in the male reproductive system, such as spermatogenesis, steroidogenesis and normal sperm cells physiology [39,40]. Nevertheless, an increased production of free radicals and peroxides in intracellular and extracellular milieu of male gonads is connected to a reduction in the quantity of sperm cells and a rise in the likelihood of abnormal spermatogenesis [41].

AgNPs-induced oxidative stress may have contributed to the changes observed in this study in steroid hormones, aromatase and steroid receptors in the testes. Previous studies have also shown that AgNPs induce reactive oxygen species and cell damage [30]. This leads to lipid peroxidation of the cell membrane [29]. The study conducted by Paciorek et al. [42] reported that the production of primary products of cellular lipid oxidation (lipid hydroperoxides) was stimulated by AgNPs and subsequently transformed into secondary lipid end-products or glutathione peroxidase substrates [43]. Our study showed an increased level of 7-KCH as a consequence of uncontrolled lipid peroxidation in testicular cells and impaired redox balance [44]. The cytotoxic and pro-oxidant effects of nanoparticles disrupt steroid hormones metabolism [3,45].

**Figure 8:**
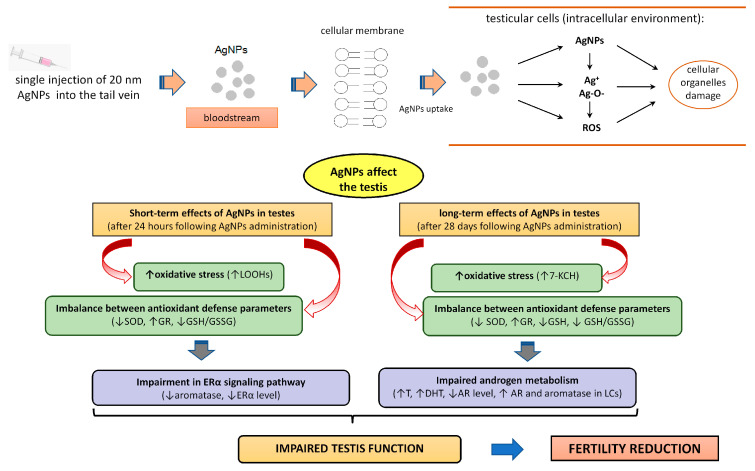
The diagram demonstrates the internalization of AgNPs in testicular cells and the mechanism behind their action in the testes. Abbreviations: 7-KCH, 7-ketocholesterol level; Ag^+^, silver ions; AR, androgen receptor; DHT, dihydrotestosterone level; ERα, estrogen receptor α; GSH, reduced glutathione level; GR, glutathione reductase activity; GSSG, oxidized form of glutathione level; LCs, Leydig cells; LOOHs, lipid hydroperoxides level; ROS, reactive oxygen species; SOD, superoxide dismutase activity; T, testosterone level; ↑, increase; ↓, decrease.

The release of Ag^+^ ions from the surface of nanoparticles is proposed to be a principal mechanism underlying the toxicity and pro-oxidative effects of AgNPs. In physiological conditions, nanosilver is recognized for inducing the “Trojan horse effect”, wherein Ag^+^ ions are released into the environment [46]. The Ag^+^ release is influenced by various factors, including the size, shape and surface coating of the nanoparticles, as well as environmental conditions such as pH and temperature. Following intravenous administration, it is probable that the effects observed are attributed to Ag ions rather than the nanoparticles, per se. In the bloodstream, AgNPs can interact with proteins and other components of biological fluids. These interactions may influence the release of silver ions from the AgNPs surface. Additionally, AgNPs may be taken up by cells, and once inside cells, they may undergo dissolution, leading to the release of silver ions [5]. The mechanism of toxic effects of AgNPs in the male reproductive system is presented in Figure 8.

## 5. Conclusions

We demonstrated that a single intravenous injection of AgNP caused adverse changes in steroid hormones metabolism. Our findings indicate that intravenous exposure to AgNPs resulted in negative effects on male gonads, including enhanced oxidative stress and impaired redox balance, leading to impairment of steroid hormone receptors in a time-depending manner. Moreover, the short-time effects of AgNPs exposure caused redox imbalance in the gonads, while the longer-time effect after nanosilver exposition was associated with impaired steroid metabolism, which most likely results from increased oxidative stress.

Although we obtained interesting results, our study also had limitations. Toxicity studies on nanosilver in in vivo models are conducted with various methodologies, including exposure time, route of administration of silver nanoparticles and their surface characteristics. In our study, we examined the effects of a single dose of 20 nm diameter nanosilver coated with BSA without comparing it to submicron scale silver particles or silver in the ionized form. Whilst the experimental design used in the study may curtail the drawing of definitive conclusions regarding potential toxicity of AgNPs in the male reproductive system, it simultaneously minimized the need for animal experimentation. The principal intention of the research focused on both short- and long-term implications exhibited by single dosages of AgNPs when administered intravenously, revealing a distinct lack of available data regarding this particular subject within the existing scientific literature.

Our research significantly enhanced the current understanding of the reproductive toxicity of silver nanoparticles used in biomedical applications and provided valuable insights. The investigation differentiated itself from previous studies by examining the time-dependent nature of the effects, evaluating outcomes at both 1 and 28 days post-intervention. This study revealed the complex connection between exposure to AgNPs and the imbalance of redox in gonadal tissues, illuminating the subtle and time-based impact of AgNPs on steroid metabolism and oxidative stress. Additionally, our study offers fresh perspectives into the time-dependent effects of AgNPs on steroid metabolism and redox balance, differentiating between short-term and long-term repercussions. This comprehension is pivotal in the improvement of current awareness on toxicity of nanomaterials. The discovered redox imbalance in the gonadal tissues after the exposure to AgNPs highlights the interdependence between nanoparticle exposure and oxidative stress, augmenting the comprehension of nanoparticle toxicity. This aspect of the research is relevant not just to toxicologists but also to researchers in areas like biochemistry and molecular biology. The significance of this study lies in its supply of detailed data which go beyond the direct aftermath of AgNPs exposure and contribute to a deeper comprehension of the complex relationship between nanomaterials, hormonal regulation and oxidative stress regarding male reproductive health.

## Figures and Tables

**Table 1:** Characterization of AgNPs in water after dispersion (mean ± SD) (modified from Lankoff et al. [14]).

Parameter	BSA-Coated AgNPs
nominal size of Ag particles [nm]	20 ± 5
dynamic light scattering [nm]	84.4 ± 3.7
polydispersity index	0.295
zeta potential [mV]	−33.6

Data are presented as mean ± SD (*n* = 3).

**Table 2:** The time-dependent changes in rats testes parameters after a single dose of AgNPs intravenous administration.

Parameters		After 1 Day	After 28 Days
		Following Administration of AgNPs (Compared to Control Groups):	
oxidative stress parameters	LOOHs concentration	↑	=
	7-KCH concentration	=	↑
anti-oxidative defense parameters	SOD activity	↓	↓
	GR activity	↑	↑
	GSH concentration	=	↓
	GSH/GSSG ratio	↓	↓
steroid hormones level and steroid receptors abundance	T	=	↑
	DHT	=	↑
	Aro protein level	↓	=
	AR protein level	=	↓
	ERα protein level	↓	=
	AR in Leydig cells	=	↑
	Aro in Leydig cells	=	↑

=, lack of significant changes; **↑**, increase; **↓**, decrease.

## Data Availability

The data underlying this article will be shared on reasonable request.
